# Hsp90 Inhibitor SNX-2112 Enhances TRAIL-Induced Apoptosis of Human Cervical Cancer Cells via the ROS-Mediated JNK-p53-Autophagy-DR5 Pathway

**DOI:** 10.1155/2019/9675450

**Published:** 2019-03-25

**Authors:** Liubing Hu, Yan Wang, Zui Chen, Liangshun Fu, Sheng Wang, Xinyue Zhang, Pengchao Zhang, Xueping Lu, Huiyang Jie, Manmei Li, Yifei Wang, Zhong Liu

**Affiliations:** ^1^Guangdong Provincial Key Laboratory of Bioengineering Medicine, Institute of Biomedicine, College of Life Science and Technology, Jinan University, Guangzhou 510632, China; ^2^Guangdong Province Key Laboratory of Pharmacodynamic Constituents of TCM and New Drugs Research, Institute of Traditional Chinese Medicine and Natural Products, College of Pharmacy, Jinan University, Guangzhou 510632, China

## Abstract

Tumor necrosis factor-related apoptosis-inducing ligand (TRAIL) is a potent cancer cell apoptosis-inducing factor that can induce apoptosis in a variety of cancer cells. However, resistance to TRAIL in cancer cells is a huge obstacle in creating effective TRAIL-targeted clinical therapies. Thus, agents that can either enhance the effect of TRAIL or overcome its resistance are needed. In this study, we combined TRAIL with SNX-2112, an Hsp90 inhibitor we previously developed, to explore the effect and mechanism that SNX-2112 enhanced TRAIL-induced apoptosis in cervical cancer cells. Our results showed that SNX-2112 markedly enhanced TRAIL-induced cytotoxicity in HeLa cells, and this combination was found to be synergistic. Additionally, we found that SNX-2112 sensitized TRAIL-mediated apoptosis caspase-dependently in TRAIL-resistant HeLa cells. Mechanismly, SNX-2112 downregulated antiapoptosis proteins, including Bcl-2, Bcl-XL, and FLIP, promoted the accumulation of reactive oxygen species (ROS), and increased the expression levels of p-JNK and p53. ROS scavenger NAC rescued SNX-2112/TRAIL-induced apoptosis and suppressed SNX-2112-induced p-JNK and p53. Moreover, SNX-2112 induced the upregulation of death-receptor DR5 in HeLa cells. The silencing of DR5 by siRNA significantly decreased cell apoptosis by the combined effect of SNX-2112 and TRAIL. In addition, SNX-2112 inhibited the Akt/mTOR signaling pathway and induced autophagy in HeLa cells. The blockage of autophagy by bafilomycin A1 or Atg7 siRNA abolished SNX-2112-induced upregulation of DR5. Meanwhile, ROS scavenger NAC, JNK inhibitor SP600125, and p53 inhibitor PFT*α* were used to verify that autophagy-mediated upregulation of DR5 was regulated by the SNX-2112-stimulated activation of the ROS-JNK-p53 signaling pathway. Thus, the combination of SNX-2112 and TRAIL may provide a novel strategy for the treatment of human cervical cancer by overcoming cellular mechanisms of apoptosis resistance.

## 1. Introduction

Tumor necrosis factor-related apoptosis-inducing ligand (TRAIL), also known as apo2 ligand, is a member of the TNF family that binds to receptors to selectively target tumor cells while sparing normal cells. As a result, TRAIL and its receptor (TRAIL-R) agonist antibodies are considered attractive candidates for use as anticancer drugs in clinical studies. TRAIL leads to the formation of the death-inducing signal complex (DISC) by interacting with death receptor 4 (DR4) and death receptor 5 (DR5), followed by binding to caspase 8. Caspase 8 is recruited to DISC to activate its proteolytic properties, which induce the activation of protease caspase 3 cascades or Bcl-2 family members, facilitating the cleavage of dead substrates, ultimately leading to apoptosis [1]. Many tumors are susceptible to TRAIL-mediated apoptosis, but the development of resistance to TRAIL is also common in many types of cancer [2, 3]. Resistance to TRAIL can result from a wide range of molecular changes: the downregulation of DR4 and DR5 expression and the upregulation of decoy receptors; the overexpression of antiapoptotic molecules, including the caspase 8 inhibitor, Fas-associated death domain-like IL-1-converting enzyme-inhibitory protein (cFLIP), inhibitors of apoptosis protein (IAP) family members, and Bcl-2 family proteins; the loss of proapoptotic proteins; and the activation of the PI3K/Akt and NF-*κ*B signaling pathways [4–8]. Bortezomib and other protease inhibitors have been found to reverse resistance to TRAIL, but have long been considered to be highly toxic [9, 10]. Therefore, there is a great need for anticancer drugs that are able to regulate cancer cell resistance to TRAIL and thus improve the therapeutic effect of TRAIL on tumors through combined treatment.

As molecular chaperones, heat shock protein 90 (Hsp90) can help intracellular signal transduction proteins fold, stabilize, and mediate cellular homeostasis, cell survival, and transcriptional regulation. It plays an important role in both normal and stress conditions and in pathological states such as cancer [11–13]. In contrast to normal cells, Hsp90 in cancer cells is usually upregulated by exposure to different kinds of stresses, such as acidosis, low oxygen tension, and nutrient deprivation [14, 15]. It has been found that the overexpression of Hsp90 was essential in protecting therapeutic agent-induced apoptosis and could be a potential target molecule for anticancer drugs [16, 17]. One of the challenges of targeted cancer therapies is that the malignant progression of cancer cells is often related to a variety of molecular abnormalities. Hsp90 inhibitors can degrade Hsp90 client proteins (including cancerogenic proteins) by targeting a single protein (Hsp90), thereby affecting several signaling pathways. Hsp90 regulates over 100 client proteins. These client proteins include Akt, Raf-1, IKK, Her2, Cdk4, MMP2, and VEGFR2 and play an important role in tumor cell survival, growth, migration, and angiogenesis [18–20]. Besides, inhibition of Hsp90 could increase ER stress to disrupt mitochondrial homeostasis and, thereby, stimulate the production of mitochondrial ROS, leading to the selective killing of cancer cells when compared to normal cells, by elevating ER stress originating from the accumulation of unfolded protein in the ER [21–23]. Meanwhile, rational drug combination of prooxidant and antioxidant might be a potential new treatment strategy to combat drug resistance in cancer [22]. In recent years, dozens of Hsp90 have been developed for oncotherapy, including first-generation Hsp90 inhibitors (geldanamycin and its derivatives) and second-generation Hsp90 inhibitors (NVP-AUY922). Geldanamycin 17-AAG (tanespimycin) is a natural product and was the first Hsp90 inhibitor to enter clinical trials [11, 12]. Hsp90 inhibitors have therapeutic applications in melanoma, AML, castrated refractory prostate cancer, NSCLC, and other types of cancers [24]. Nevertheless, there are no FDA-approved Hsp90 inhibitors or standardized experiments that confirm the inhibitory effect of Hsp90. The most significant off-target toxicity of geldanamycin derivatives in clinical use is their hepatotoxicity caused by the benzoquinone moiety. In addition to hepatotoxicity, the ocular and cardiac toxicity of these drugs limits their further clinical development [24, 25].

Geldanamycin or 17-AAG inhibition of Hsp90 has been reported to sensitize some cells to TRAIL-induced apoptosis [26, 27]. The combination of 17-AAG and death receptors targeting agent TRAIL can synergistically increase antitumor activity and eliminate resistance to TRAIL in gliomas [28]. In TRAIL-resistant tumor cell lines, such as prostate LNCaP cells, colon cancer HT29, and RKO cells, pre- or coexposure to 17-AAG with TRAIL can induce high levels of cell apoptosis [29–31]. Studies have shown that the combination of 17-AAG with TRAIL coinduces apoptosis as a result of the deregulation of NF-*κ*B or Akt cell survival pathways [32, 33].

We have previously proven that SNX-2112 was a novel, highly efficient, and highly selective small molecule inhibitor of Hsp90 that could competitively integrate with the N-terminal ATP-binding site of Hsp90 and exhibit anticancer activity in cancer cells, including breast cancer [34], multiple myeloma [35], melanoma [36, 37], chronic myeloid leukemia [38], esophageal cancer [39], and head and neck squamous cell carcinomas [40]. Especially in human hepatocellular carcinoma cells, SNX-2112 could induce apoptosis, elevating the role of ER stress [41]. In this study, we attempted to use SNX-2112 in combination with TRAIL to overcome the resistance of cervical cancer HeLa cells to TRAIL-induced cell apoptosis and provided a new therapeutic strategy for the clinical treatment of cervical cancer.

## 2. Materials and Methods

### 2.1. Cell Culture

Human cervical cancer cells (HeLa, SiHa, and Caski) were purchased from the Cell Bank of the Chinese Academy of Sciences (Shanghai, China), stored in liquid nitrogen, and cultured in RPMI-1640 or DMEM medium supplemented with 10% FBS and 100 U/mL penicillin/streptomycin in 5% CO_2_ at 37°C.

### 2.2. Reagents and Antibodies

SNX-2112 was synthesized as previously described in our lab with >98.0% purity [42], dissolved in dimethyl sulfoxide (DMSO) to obtain a 100 mM stock solution, and stored at −20°C. TRAIL was purchased from Merck Millipore (Waltham, MA, USA). N-Acetyl-cysteine (NAC) and 2′,7′-dichlorofluorescein diacetate (DCFH-DA) were purchased from the Beyotime Institute of Biotechnology (Shanghai, China). Bafilomycin A1 (BFA) was purchased from Selleck Chemicals (Shanghai, China). Dimethyl sulfoxide (DMSO) and 3-(4,5-dimethylthiazol-2-yl)-2,5-diphenyltetrazolium bromide (MTT) were purchased from Sigma (St. Louis, MO, USA). Antibodies for GAPDH, Bcl-2, Bcl-xL, FLIP, pro-caspase 3, cleaved-caspase 3 (c-caspase 3), cleaved-caspase 8 (c-caspase 8), cleaved-PARP (c-PARP), Akt, p-Akt (Ser473), DR4, DR5, LC3, Beclin1, Atg7, p62, p-mTOR, p-S6, p-4EBP1, p53, p-ERK, ERK, p-p38, p38, p-JNK, and JNK were purchased from Cell Signaling Technology (CST; Beverly, MA, USA).

### 2.3. Cell Viability Assay

Cell viability was evaluated using the MTT assay. Exponentially growing cells were plated in 96-well culture plates (5000/well in 100 *μ*L medium) for 24 h and incubated with a series of concentrations of SNX-2112 (0-1 *μ*M) or TRAIL (0-1000 ng/mL) for 48 h. A 10 *μ*L volume of MTT working solution was added to each well for 4 h. After the medium was removed, formazan crystals solubilized in 100 *μ*L DMSO/well, and the absorbance values were assessed at 570 nm on a microplate reader (Bio-Rad, Hercules, CA, USA). The inhibition ratio was calculated as follows: (*A* control – *A* treated)/*A* control × 100%, where *A* treated and *A* control are the absorbance of the treated and control groups after a 48 h incubation, respectively.

### 2.4. Synergy Analysis

The synergistic interaction of SNX-2112 and TRAIL was calculated by using the Chou-Talalay method [43–45]. The combination index (CI) was evaluated using CompuSyn software (ComboSyn Inc., Paramus, NJ, USA) for each combination. A fitting curve of CI value data points was plotted according to the data generated by the viability assay using CompuSyn software. When the fitting curve of the drug's CI value was near or on the additive line (CI value = 1), it represented an additive treatment effect. The fitting curve below or above the additive line represents the synergism or antagonism effect, respectively.

### 2.5. ROS Detection

Generation of intracellular ROS was detected using DCFH-DA, a dye that detects reactive oxygen species (ROS). After cell treatment, HeLa cells were stained with the DCFH-DA (20 *μ*M) fluorescent dye for 20 min. Fluorescence was measured with an epifluorescence microscope (Nikon, Japan).

### 2.6. Western Blotting Analysis

To assess the effect of SNX-2112 on TRAIL-induced protein activation, HeLa cells were collected and lysed in sodium dodecyl sulfate (SDS) buffer for 30 min at 4°C. After centrifugation at 12000 g for 20 min, the supernatant was pipetted for analysis with a bicinchoninic acid assay. Equivalent amounts (>30 *μ*g) of protein were separated by 10-15% SDS-PAGE and transferred onto polyvinylidene difluoride membranes. Membranes were blocked in 5% skimmed milk dissolved in Tris-buffered saline (TBS) containing 0.1% Tween 20 (TBST) at room temperature for 1 h. Membranes were then incubated with the corresponding primary antibody (1 : 1000) at 4°C overnight, then washed in TBST and probed with secondary antibody (1 : 6000-1 : 8000) in TBST for 2 h at room temperature. Protein expression was detected using an enhanced chemiluminescence kit (4 A Biotech Co. Ltd., Beijing, China). GAPDH was used as a loading control.

### 2.7. Flow Cytometry for Apoptosis Measurement

Cell apoptosis was quantified by flow cytometry using an Annexin V-FITC/propidium iodide (PI) staining kit (Beyotime Institute of Biotechnology). Cells were seeded at 1.0 × 10^5^ cell/mL in 6-well plates for 24 h, then exposed to the indicated concentrations of SNX-2112 or TRAIL alone or in combination for 48 h. Cell samples were prepared according to the manufacturer's instructions. In brief, cells were washed and resuspended in 500 *μ*L PBS, then incubated with 10 *μ*L PI and 10 *μ*L Annexin V-FITC at room temperature in the dark for 15 min. Data acquisition and analysis were performed using a FACSCalibur flow cytometer with CellQuest software (Becton-Dickinson, Mississauga, CA, USA).

### 2.8. Immunofluorescence Analysis of the Expression of Cell Surface DR5

To detect the cellular expression of DR5, HeLa cells plated in chamber slides were treated with the indicated concentrations of SNX-2112/TRAIL for 48 h at 37°C. Samples were processed as described before [46]. Briefly, after fixing, permeabilizing, and blocking, the cells were incubated for 2 h at room temperature with an antibody against DR5 (1 : 50, Cell Signaling Technology). The cells were then probed with an Alexa Fluor 594-conjugated anti-rabbit antibody (1 : 1000) for 1 h. Nuclei were stained with DAPI (1 mg/mL, Sigma-Aldrich) for 15 min. Finally, cells were washed, and fluorescence was examined by laser scanning confocal microscopy (Nikon, Japan).

### 2.9. Transfection of siRNA

Transfection of HeLa cells was conducted with siRNA transfection reagent of jetPRIME (Polyplus, New York, NY, USA) following the manufacturer's instructions. High-purity controls (scrambled RNA), along with DR5, DR4, and Atg7, were obtained from GenePharma. The targeting sequences of the siRNA constructs were DR5 siRNA, 5′-UUCUGGGAACACGAGCAACAG-3′; DR4 siRNA-1, 5′-AAGAACCA GCAGAGGUCACAA-3′; DR4 siRNA-2, 5′-CACCAAUGCUUCCAACAAUdTdT-3′; Atg7 siRNNA-1, 5′-CCCUGUACUCCUCAACAAG-3′; and Atg7 siRNA-2, 5′-GCCUCUCUAUGAGUUUGAA-3′.

### 2.10. Detection of JC-1

HeLa cells (2 × 10^5^) were seeded in 60 mm Petri dishes for a day before the experiment. After treatment with SNX-2112 alone or in combination with TRAIL at the indicated concentrations for 48 h, cells were harvested, washed twice with ice-cold PBS, and incubated with JC-1 (10 *μ*g/mL) in the dark for 15 min at 37°C. Cells were then washed three times with ice-cold PBS and analyzed by flow cytometry using emission wavelengths of 590 nm and 525 nm.

### 2.11. Statistical Analysis

All data were expressed as the mean ± SD, and three independent experiments were performed to confirm the reliability of the data. For groups of two, statistical analysis was carried out by using two-tailed unpaired Student's *t* test. For groups of three or more, comparison was carried out using one-way ANOVA multiple. *P* values <0.05 and <0.01 were considered as statistically significant.

## 3. Results

### 3.1. SNX-2112 and TRAIL Synergistically Induce Cytotoxicity in Cervical Cancer HeLa Cells

**Figure 1:**
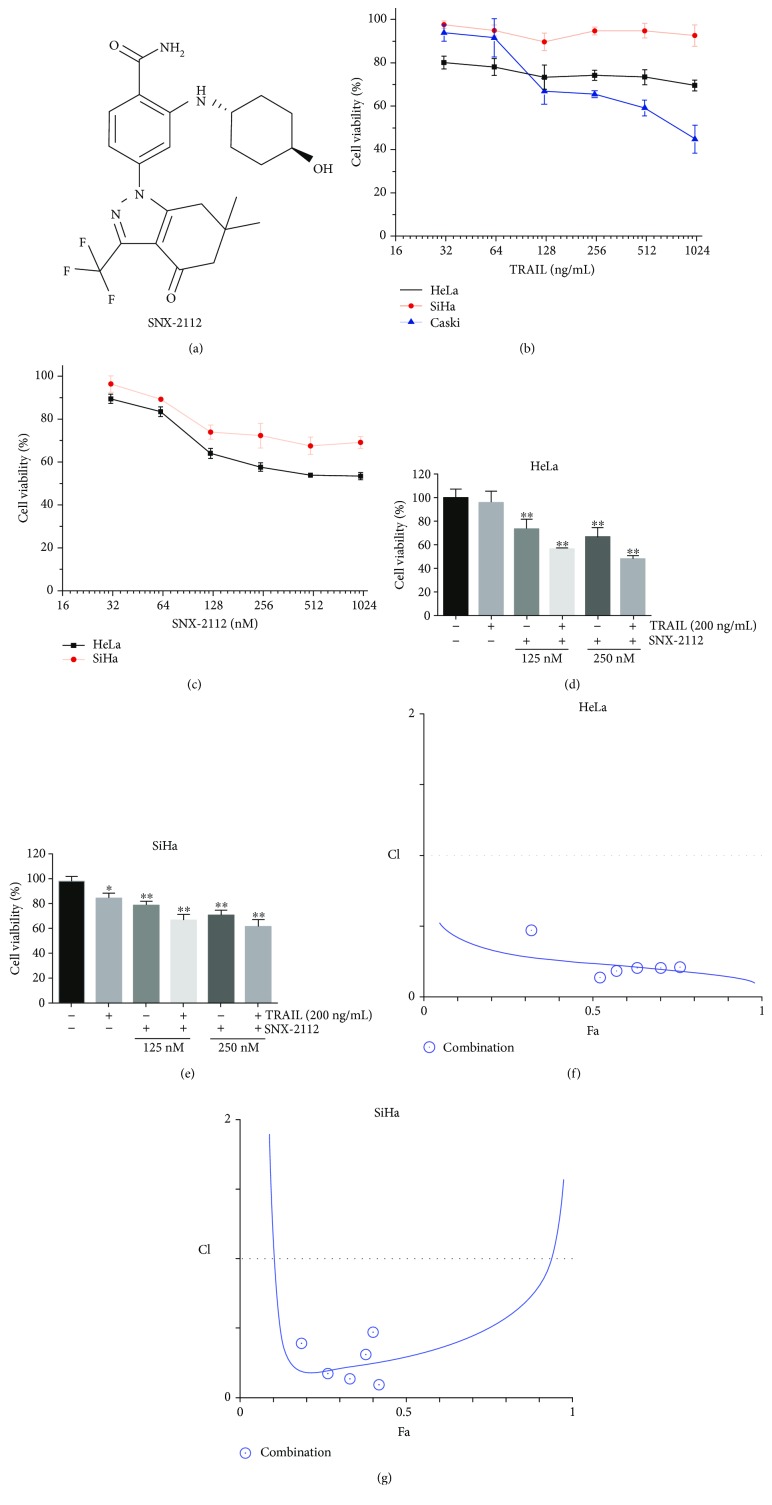
SNX-2112 enhances TRAIL-induced cytotoxicity in human cervical cancer cells. (a) Chemical structure of SNX-2112. (b) Cervical cancer cell lines, HeLa, SiHa, and Caski, were treated with TRAIL at different concentrations (0, 31.25, 62.5, 125, 250, 500, and 1000 ng/mL) for 48 h. Cell viability was assessed by MTT assay. (c) Cervical cancer cell lines, HeLa and SiHa, were treated with SNX-2112 at different concentrations (0, 31.25, 62.5, 125, 250, 500, and 1000 nM) for 48 h. Cell viability was assessed by MTT assay. (d) HeLa or (e) SiHa cells were treated with either TRAIL (200 ng/mL) or SNX-2112 (125, 250 nM) or in combination for 48 h. Cell viability was assessed by MTT assay. (f-g) Fitting curves of CIs for TRAIL and SNX-2112 combination in SiHa and HeLa cell lines plotted and calculated using CompuSyn software. Data are represented as mean ± SD. Error bars represent SD from three separate experiments. ^∗^ *P* < 0.05 and ^∗∗^ *P* < 0.01 compared with the control group.

To investigate whether SNX-2112 could synergize with TRAIL to suppress human cervical cancer cell viability, a range of cervical cancer cell lines, including HeLa, SiHa, Caski cells, were tested. Before testing the combined effect of SNX-2112 and TRAIL therapy, we first evaluated the cytotoxicity of TRAIL monotherapy in three human cervical cancer cell lines by means of a MTT assay. Our data showed that, at concentrations of 1000 ng/mL or lower, TRAIL showed no significant antitumor effect on HeLa and SiHa cells, indicating that both cervical cell lines either had low sensitivity or were resistant to TRAIL monotherapy (Figure 1(b)). Both types of cervical cells were assessed with SNX-2112 monotherapy, and similar results were found (Figure 1(c)). Meanwhile, when the cells were cotreated with 125 or 250 nM of SNX-2112 and 200 ng/mL of TRAIL for 48 h, cell viability of both cell lines was markedly inhibited (Figures 1(d) and 1(e)).

For the two cell lines, HeLa and SiHa cells, the combination of both agents within a certain concentration range was the most effective treatment for blocking cell growth rather than the use of either agent alone. To determine the effect of a combined treatment of SNX-2112 and TRAIL, a combination study was performed. We obtained the fitting curves of CI value data points, and a fitted curve analysis was performed using CompuSyn software to define the interactions between SNX-2112 and TRAIL in HeLa and SiHa cells. According to the Chou–Talalay method, CIs were always less than 1 for the combination treatments in HeLa cell lines, indicating that cotreatment of SNX-2112 and TRAIL led to synergistic inhibitory effects on cell proliferation in HeLa cell lines, but not in SiHa cell lines (Figures 1(f) and 1(g)). Based on the above observations, we concluded that SNX-2112 markedly enhanced TRAIL-induced cytotoxicity in human cervical cancer cells and that the combination was considered synergistic in HeLa cells.

### 3.2. SNX-2112 Sensitizes TRAIL-Mediated Apoptosis via the Activation of Caspases in TRAIL-Resistant HeLa Cells

**Figure 2:**
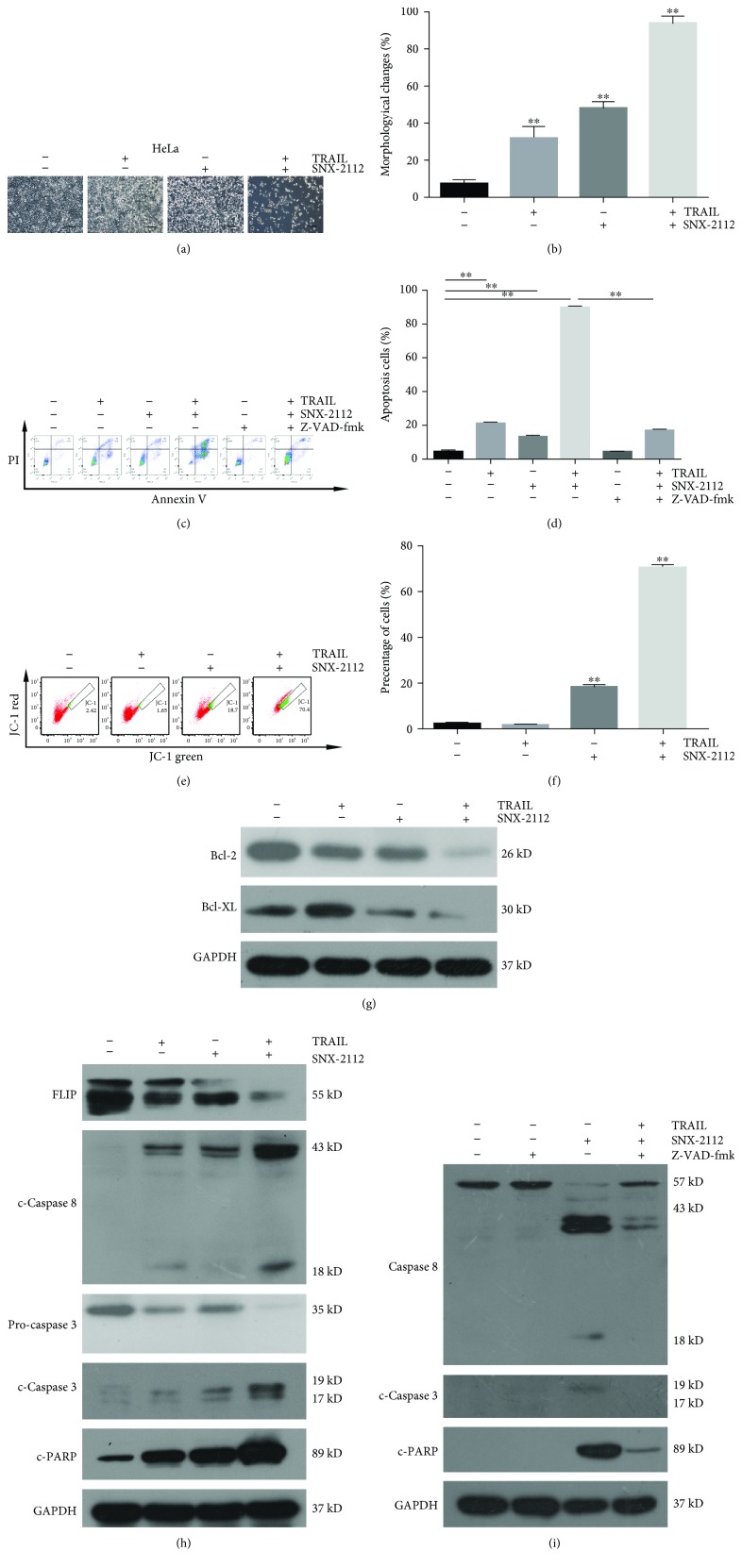
SNX-2112 is synergistic to TRAIL and coinduces HeLa cell apoptosis. (a-i) Cells were treated with either TRAIL (200 ng/mL) or SNX-2112 (125 nM) alone or in combination for 48 h. Using FACS analysis assays, the cells were pretreated with or without 20 *μ*M Z-VAD-fmk for 1 h. (a, b) Microscopic cell morphologies. Scale bar: 250 *μ*m. Morphologically altered cells were counted and analyzed. (c, d) The cells were stained with Annexin V and propidium iodide (PI), followed by FACS analysis. (e, f) FACS analysis of Δψ_m_ by JC-1 staining. After treatment with drugs, cells were stained with JC-1 for 20 min. (g h) The Bcl-2, Bcl-XL, FLIP, pro-caspase 3, c-caspase 3 and 8, and c-PARP proteins were analyzed by Western blotting. (i) Apoptosis-associated proteins (c-caspase 3 and 8 and c-PARP) were detected in the presence of Z-VAD-fmk by Western blotting. GAPDH was used as a protein loading control. Data are represented as mean ± SD. Error bars represent SD from three separate experiments. ^∗^ *P* < 0.05 and ^∗∗^ *P* < 0.01 compared with the control group.

To investigate whether SNX-2112 could sensitize HeLa cancer cells to TRAIL-mediated apoptosis, HeLa cells were initially treated with 125 nM SNX-2112 or 200 ng/mL TRAIL alone or in combination for 48 h. Their morphology was found to be significantly affected by the combined treatment compared with the control or monotherapy group (Figures 2(a) and 2(b)). Apoptosis characteristics, including cell shrinkage, apoptotic bodies, and detachment from the plate, were observed more generally in HeLa cells treated with both SNX-2112 and TRAIL (Figure 2(a)). Furthermore, the results of flow cytometry analysis for apoptosis showed that SNX-2112 or TRAIL alone induced 21.4 or 13.5% apoptosis, individually, and that the combination of treatment with SNX-2112 and TRAIL augmented apoptosis to 90.0%. Nevertheless, the synergistic effect of this treatment was abolished by pretreatment with Z-VAD-fmk, the cell-permeable pan caspase inhibitor (Figures 2(c) and 2(d)). Change in the mitochondrial membrane potential (Δ*ψ*_m_) is an early event preceding caspase activation and is regarded as a hallmark of apoptosis [47]. Therefore, we measured Δ*ψ*_m_ in TRAIL/SNX-2112-treated HeLa cells using the membrane-permeable JC-1 dye. As shown in Figures 2(e) and 2(f), treatment with TRAIL and SNX-2112 induced a marked increase in JC-1-related green fluorescence in HeLa cells.

To investigate the mechanisms behind the role of SNX-2112 in the intensification of TRAIL-induced apoptosis, we quantified multiple cell death pathway components that could be influenced by SNX-2112. The results indicated that the expression of antiapoptotic Bcl-2 and Bcl-XL was suppressed (Figure 2(g)). As single agents, SNX-2112 or TRAIL reduced the expression level of FLIP and induced low levels of caspase 3 and 8 and poly (ADP-ribose) polymerase (PARP) cleavage, whereas their combination induced profoundly higher processing of these proteins (Figure 2(h)). Furthermore, consistent with the flow cytometry analysis, the upregulation of apoptosis-associated proteins (c-caspase 3 and 8 and c-PARP) was abolished by pretreatment with Z-VAD-fmk (Figure 2(i)). At least in part, these results may help to clarify the synergistic effect. Taken together, these experimental data suggested that SNX-2112 could sensitize TRAIL-mediated apoptosis via the activation of caspases in TRAIL-resistant HeLa cells.

### 3.3. SNX-2112-Induced Accumulation of ROS Is Pivotal for SNX-2112-Stimulated TRAIL-Induced Apoptosis

**Figure 3:**
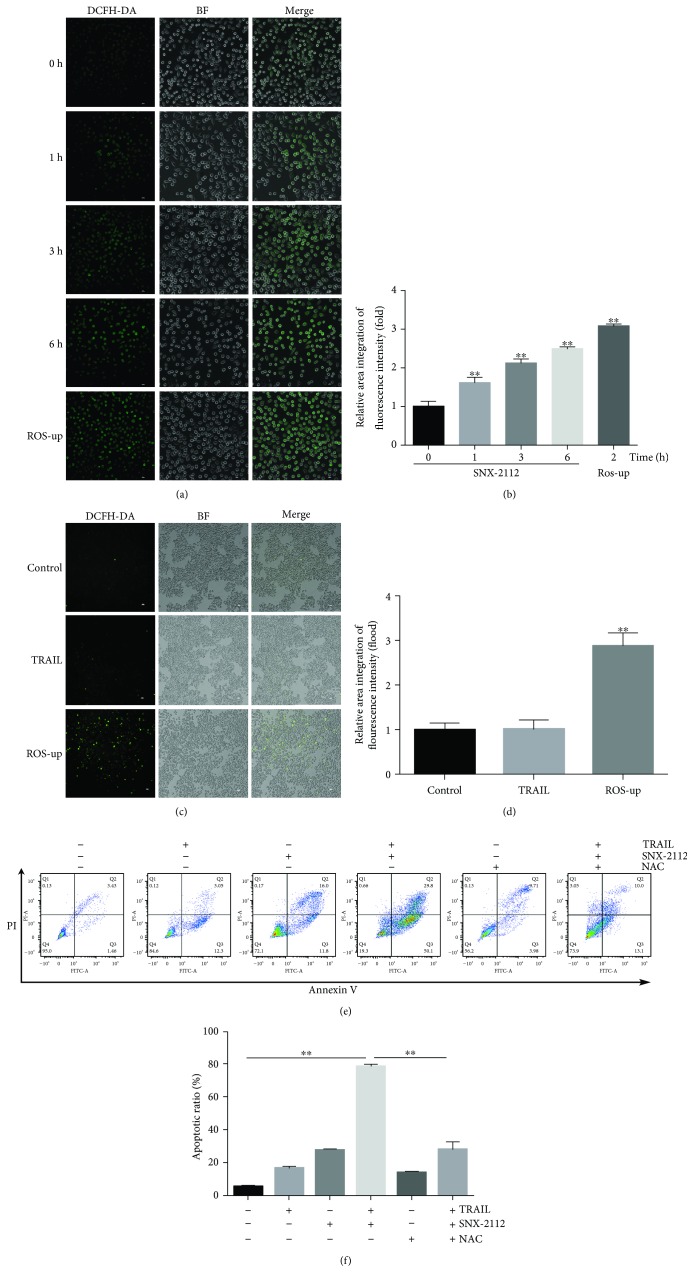
SNX-2112-induced accumulation of ROS significantly enhances apoptosis induced by TRAIL. HeLa cells were treated with (a, b) SNX-2112 (125 nM) for the indicated time (0, 1, 3, and 6 h) or (c, d) TRAIL (200 ng/mL) for 6 h. ROS-up was used to be the positive control. ROS levels were measured with an epifluorescence microscope by DCFH-DA staining. (e, f) HeLa cells were pretreated with or without 20 mM NAC for 2 h and then treated with or without TRAIL/SNX-2112 for 48 h. The cells were stained with Annexin V/PI and TRAIL/SNX-2112-induced apoptosis was analyzed by flow cytometry. Data are represented as mean ± SD. Error bars represent SD from three separate experiments. ^∗^ *P* < 0.05 and ^∗∗^ *P* < 0.01 compared with the control group.

To investigate whether ROS contributed to SNX-2112-enhanced TRAIL-induced apoptosis, we measured the level of ROS in HeLa cells. In the DCFH-DA-based fluorescent assay, HeLa cells were treated with SNX-2112, and green fluorescence was found to markedly increase in a time-dependent manner (Figures 3(a) and 3(b)), suggesting the production of ROS in HeLa cells. However, we found that treatment with TRAIL alone could not increase the level of ROS in HeLa cells (Figures 3(c) and 3(d)).

Notably, flow cytometry analysis showed that pretreatment with NAC, a ROS scavenger, significantly rescued SNX-2112/TRAIL-induced apoptosis in HeLa cells (Figures 3(e) and 3(f)). Sensitization to apoptosis was completely abolished by NAC. These results suggest that SNX-2112-induced ROS plays a key role in apoptosis induced by treatment with a combination of SNX-2112 and TRAIL.

### 3.4. DR5 Upregulation Mediates SNX-2112-Stimulated TRAIL-Induced Apoptosis

**Figure 4:**
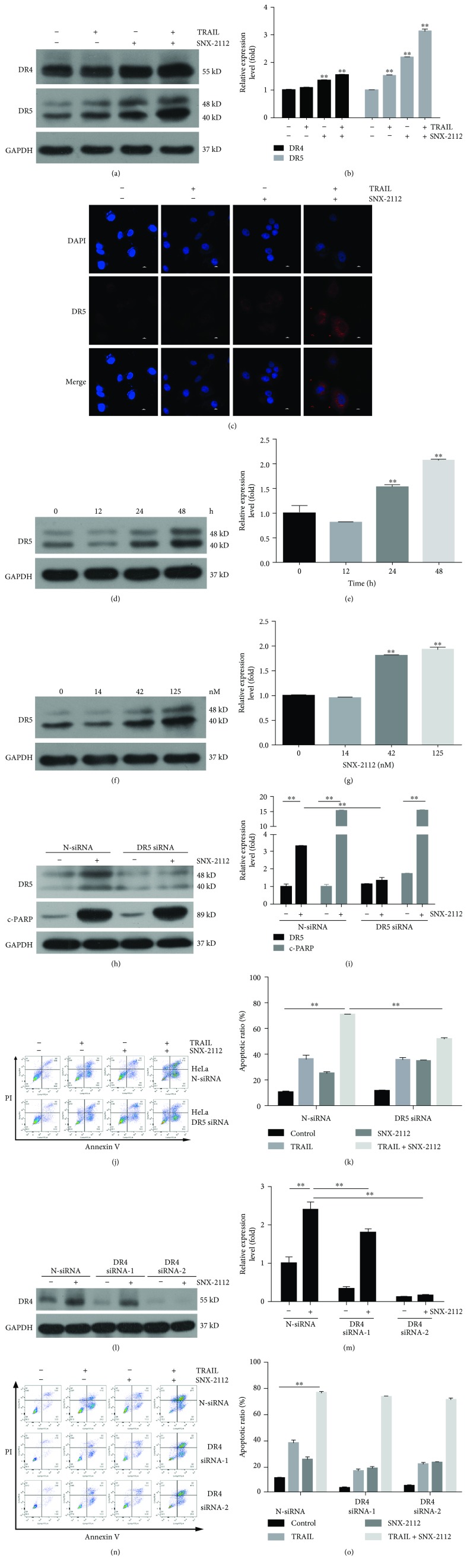
SNX-2112-induced DR5 mediates the combined effect of TRAIL and SNX-2112. HeLa cells were treated with SNX-2112 (125 nM) in either the absence or the presence of TRAIL (200 ng/mL) for 48 h. (a, b) Western blotting was performed to detect the levels of DR4 and DR5. Densitometry analyses of the bands for each protein were performed. (c) Immunofluorescence analyses were carried out using the indicated antibody (DR5) to detect the cellular expression of DR5. Scale bar: 10 *μ*m. (d, e) HeLa cells were treated with SNX-2112 (125 nM) for the indicated time (12, 24, and 48 h). The protein levels of DR5 in whole cell lysates were determined using the specific antibody. Densitometry analyses of the bands for the protein were performed. (f, g) HeLa cells were treated with SNX-2112 at different concentrations (0, 14, 42, and 125 nM) for 48 h. The protein levels of DR5 in whole cell lysates were determined using the specific antibody. Densitometry analyses of the bands for the protein were performed. (h, i) HeLa cells were transfected with control siRNA (N-siRNA) or DR5 siRNAs. After treatment with SNX-2112 (125 nM) for 48 h, Western blotting was used to analyze whole-cell extracts. The protein levels of DR5 and c-PARP were determined using the specific antibody. Densitometry analyses of the bands for each protein were performed. (j, k) The resultant cells were exposed to SNX-2112 (125 nM) in either the absence or the presence of TRAIL (200 ng/mL) for 48 h. Cells were stained with Annexin V/PI, and cell death was measured by FACS. (l, m) HeLa cells were transfected with control siRNA (N-siRNA) or DR4 siRNAs. After treatment with SNX-2112 (125 nM) for 48 h, Western blotting was used to analyze whole-cell extracts. The protein levels of DR4 were determined using the specific antibody. Densitometry analyses of the bands for each protein were performed. (n, o) The resultant cells were exposed to SNX-2112 (125 nM) in either the absence or the presence of TRAIL (200 ng/mL) for 48 h. Cells were stained with Annexin V/PI, and cell death was measured by FACS. Data are represented as mean ± SD. Error bars represent SD from three separate experiments. ^∗^ *P* < 0.05 and ^∗∗^ *P* < 0.01 compared with the control group.

Two TRAIL receptors, DR4 and DR5, are known to trigger apoptotic signals upon TRAIL binding via the functional death domain. In certain cancer cell lines, reduced expression of TRAIL receptors DR4 and DR5 and/or upregulation of the decoy receptors DcR1 and DcR2 have been defined as the main causes of TRAIL resistance [48]. To further explore the molecular mechanisms underlying ROS-mediated TRAIL sensitization in HeLa cells, we investigated whether SNX-2112-induced TRAIL receptor upregulation and its subsequent increased expression contributed to synergistic apoptosis caused by the combination of SNX-2112 and TRAIL. As expected, our Western blotting results showed that SNX-2112 alone or in combination with TRAIL obviously upregulated DR5 in HeLa cells. Although the expression of DR4 was also significantly increased (Figures 4(a) and 4(b)), the upregulation of DR5 was predominant compared to DR4. As a result, in subsequent experiments, we focused on DR5. Consistent with the changes observed in the protein, immunofluorescence analysis showed that SNX-2112 treatment increased DR5 cell surface expression (Figure 4(c)). Subsequently, we found that the treatment of HeLa cells with SNX-2112 could significantly induce increased protein levels of DR5 in both concentration-dependent and time-dependent manners (Figures 4(d)-4(g)).

To determine the role of DR5 and DR4, we applied a specific siRNA to silence DR5 and DR4 expression. Western blotting revealed that SNX-2112-induced upregulation of DR5 was abolished by DR5 siRNA (Figures 4(h) and 4(i)). Flow cytometry analysis showed that the silencing of DR5 significantly decreased the apoptosis induced by TRAIL/SNX-2112 (Figures 4(j) and 4(k)). However, cleaved-PARP induced by SNX-2112 was not affected by DR5 siRNA (Figure 4(h)). On the other hand, Western blotting also showed that SNX-2112-induced upregulation of DR4 was abolished by DR4 siRNAs (Figures 4(l) and 4(m)). However, flow cytometry analysis showed that the silencing of DR4 did not affect the apoptosis induced by TRAIL/SNX-2112 (Figures 4(n) and 4(o)). These results suggest that DR5 upregulation significantly enhanced apoptosis induced by treatment with a combination of SNX-2112 and TRAIL.

### 3.5. SNX-2112 Can Significantly Induce Autophagy to Enhance Apoptosis by Inhibiting the Akt/mTOR Signaling Pathway in HeLa Cells

By the downregulation of DR4 and DR5 surface expression, the accumulation of autophagosomes in TRAIL-resistant breast cancer cells induces TRAIL resistance [49]. We studied whether autophagy was related to the mechanism that accounted for the upregulation of SNX-2112-induced DR5. The turnover of LC3 protein, a characteristic autophagosomal marker, from the cytosolic form LC3-I to the lipidated form LC3-II is associated with the formation of autophagosomes [50]. Beclin1 is a pivotal protein in autophagy, whose upregulation can lead to autophagy [51].

**Figure 5:**
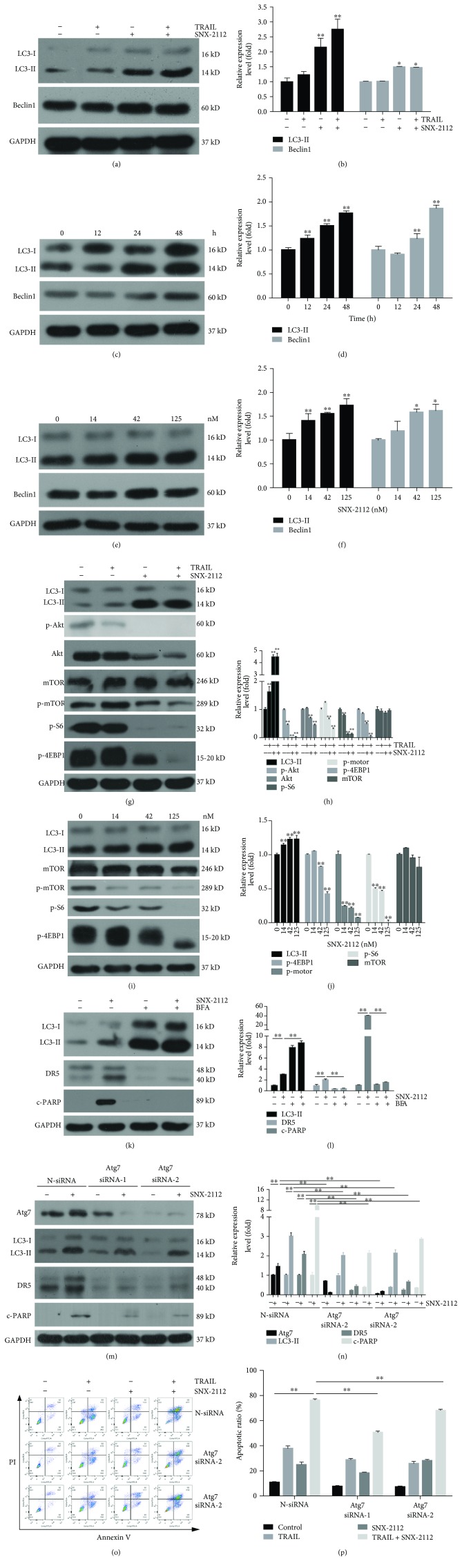
DR5 expression is regulated by SNX-2112-induced autophagy. (a, b) HeLa cells were treated with either TRAIL (200 ng/mL) or SNX-2112 (125 nM) alone or in combination for 48 h. Western blotting was performed to detect the levels of LC3 and Beclin1. Densitometry analyses of the bands for each protein were performed. (c-f) HeLa cells were treated with SNX-2112 for the indicated time (12, 24, and 48 h) or concentration (0, 14, 42, and 125 nM). Western blotting was performed to detect the levels of LC3 and Beclin1. Densitometry analyses of the bands for each protein were performed. (g, h) HeLa cells were treated with SNX-2112 (125 nM) alone or in combination with TRAIL (200 ng/mL) for 48 h. Western blotting was performed to detect the levels of LC3, p-Akt, Akt, p-mTOR, mTOR, p-S6, and p-4EBP1. Densitometry analyses of the bands for each protein were performed. (i, j) HeLa cells were treated with SNX-2112 for the indicated concentration (0, 14, 42, and 125 nM). Western blotting was performed to detect the levels of LC3, p-mTOR, mTOR, p-S6, and p-4EBP1. Densitometry analyses of the bands for each protein were performed. (k, l) HeLa cells were pretreated with BFA (50 nM) for 2 h and then treated with SNX-2112 for 48 h. LC3, DR5, and c-PARP were detected by Western blotting. Densitometry analyses of the bands for each protein were performed. (m, n) HeLa cells were transfected with control siRNA (N-siRNA) or Atg7 siRNAs. After treatment with SNX-2112 (125 nM) for 48 h, Western blotting was used to analyze whole-cell extracts. The protein levels of Atg7, LC3, DR5, and c-PARP were determined using the specific antibody. Densitometry analyses of the bands for each protein were performed. (o, p) The resultant cells were exposed to SNX-2112 (125 nM) in either the absence or the presence of TRAIL (200 ng/mL) for 48 h. Cells were stained with Annexin V/PI, and cell death was measured by FACS. Data are represented as mean ± SD. Error bars represent SD from three separate experiments. ^∗^ *P* < 0.05 and ^∗∗^ *P* < 0.01 compared with the control group.

We determined the expression levels of LC3 and Beclin1 treated with SNX-2112 alone or in combination with TRAIL for 48 h in HeLa cells. First, the combination of TRAIL and SNX-2112 was found to markedly upregulate LC3-II and Beclin1 (Figures 5(a) and 5(b)). Additionally, the autophagy markers were dramatically upregulated by SNX-2112 alone in other treated groups (Figures 5(c)-5(f)). This indicates that SNX-2112 can markedly induce autophagy in HeLa cells in a concentration- and time-dependent manner. Moreover, as shown in Figures 5(g) and 5(h), SNX-2112 was found to inhibit the expression of Akt, a client protein of Hsp90. Subsequent testing showed that Akt/mTOR signaling protein, p-Akt, p-mTOR, p-4EBP1, and p-S6 were inhibited by SNX-2112 alone or in combination with TRAIL in HeLa (Figures 5(g)-5(j)). These results suggest that the autophagy effect of SNX-2112 is a result of the inhibition of the Akt/mTOR signaling pathway in HeLa cells.

Subsequently, an autophagy inhibitor, bafilomycin A1 (BFA), was used to determine the correlation between autophagy and DR5 expression. BFA is an inhibitor of vacuolar ATPase that prevents fusion between lysosomes and autophagosomes [52]. The accumulation of LC3-II induced by SNX-2112 was found to increase in HeLa cells that had been pretreated with BFA at a concentration of 50 nM for 2 h (Figures 5(k) and 5(l)). These results indicate that BFA inhibits the degradation of LC3-II, thus blocking the autophagy. As expected, subsequently, this resulted in the downregulation of SNX-2112-induced DR5 and cleaved PARP (Figures 5(k) and 5(l)). Furthermore, we applied two specific siRNAs to silence Atg7 expression, a protein marker for autophagy. Western blotting demonstrated that SNX-2112-induced upregulation of Atg7, LC-II, DR5, and c-PARP was abolished by Atg7 siRNAs (Figures 5(m) and 5(n)). Flow cytometry analysis showed that the silencing of Atg7 significantly decreased the apoptosis induced by TRAIL/SNX-2112 (Figures 5(o) and 5(p)). These results suggest that autophagy induced by SNX-2112 is dependent on Akt/mTOR signaling and plays an important role in improving the expression of DR5 but also affects SNX-2112-induced apoptosis in HeLa cells.

### 3.6. SNX-2112 Increases p53 Expression to Enhance TRAIL-Induced Apoptosis

DR5 is a p53-regulated DNA damage-inducible cell death receptor. p53 is an important protein for the regulation of DR4 and DR5 expression at the transcriptional level. Some agents have been found to induce p53-dependent transcription of DR5 to synergize with TRAIL [53, 54].

**Figure 6:**
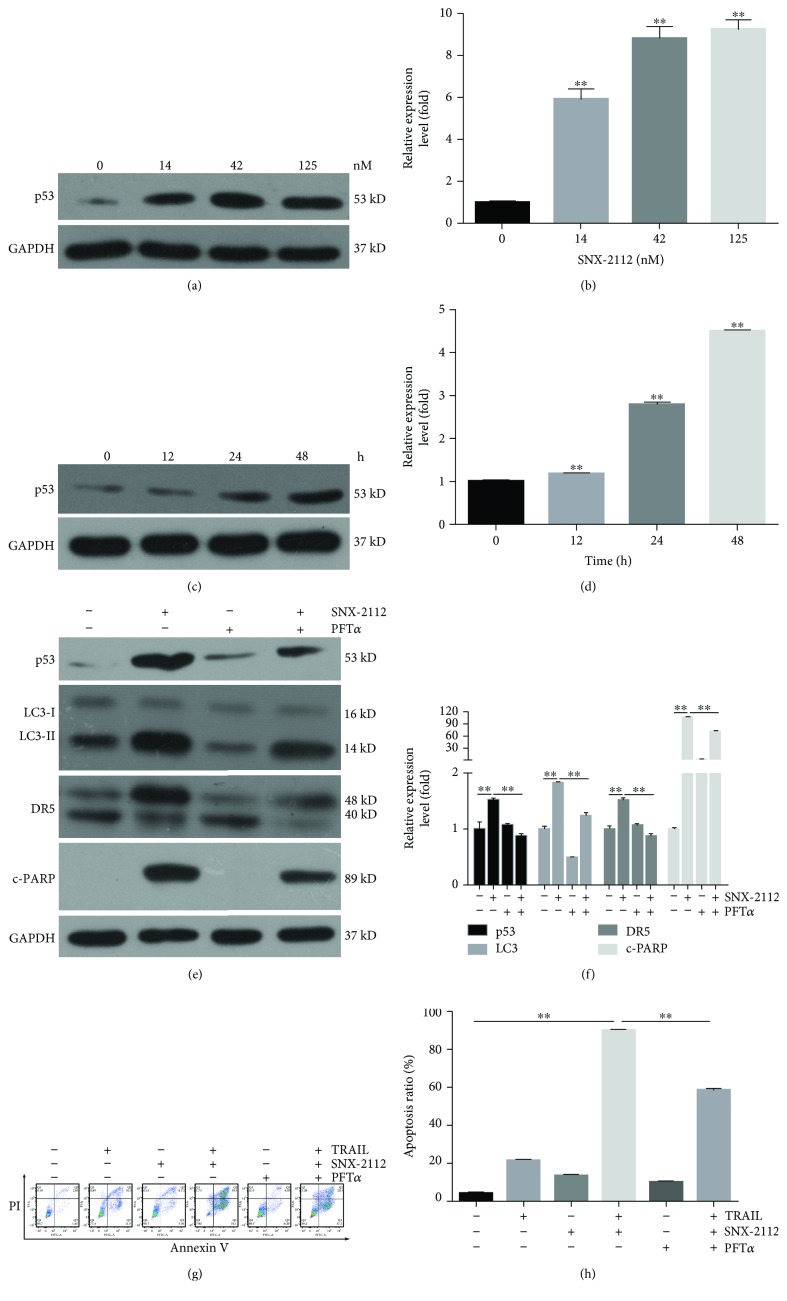
SNX-2112-induced p53 expression enhances apoptosis induced by TRAIL. (a, b) HeLa cells were exposed to SNX-2112 at the indicated dose for 48 h. Western blotting was performed to detect the levels of p53. Densitometry analyses of the bands for the protein were performed. (c, d) HeLa cells were exposed to SNX-2112 (125 nM) at the indicated time intervals. Western blotting was performed to detect the levels of p53. Densitometry analyses of the bands for the protein were performed. (e, f) HeLa cells were pretreated with the p53 inhibitor PFT*α* (20 *μ*M) and then exposed to SNX-2112 at the indicated doses. Western blotting was performed to detect the levels of p53, LC3, DR5, and c-PARP. Densitometry analyses of the bands for each protein were performed. (g, h) HeLa cells were pretreated with the p53 inhibitor PFT*α* (20 *μ*M) and then treated with either TRAIL (200 ng/mL) or SNX-2112 (125 nM) alone or in combination for 48 h. Apoptosis induced by TRAIL and SNX-2112 was measured by FACS. Data are represented as mean ± SD. Error bars represent SD from three separate experiments. ^∗^ *P* < 0.05 and ^∗∗^ *P* < 0.01 compared with the control group.

To investigate whether p53 contributed to SNX-2112-induced DR5 upregulation, we treated HeLa cells with SNX-2112 and examined p53 levels by Western blotting. As shown in Figures 6(a)-6(d), the expression of p53 was increased in a time-dependent and concentration-dependent manner by SNX-2112. Furthermore, we found that SNX-2112-induced p53 was inhibited by PFT*α*, a p53-specific inhibitor. Moreover, DR5 expression induced by SNX-2112 was abolished in these p53-inhibited HeLa cells (Figures 6(e) and 6(f)), indicating a p53-dependent mechanism. Here, we were particularly interested in the relationship between autophagy and p53. Interestingly, p53 inhibition was correlated with the downregulated expression of LC3-II (Figures 6(e) and 6(f)). Furthermore, the results of the flow cytometry assay showed that PFT*α* significantly reduced apoptosis induced by TRAIL and SNX-2112 (Figures 6(g) and 6(h)). These results suggest that SNX-2112-induced DR5 is p53-dependent and regulated through the p53-autophagy pathway. Additionally, we found that SNX-2112-induced c-PARP was abolished by PFT*α* (Figures 6(e) and 6(f)), suggesting that p53 also regulates the apoptosis induced by SNX-2112 alone. In summary, the apoptotic effect induced by the combination of SNX-2112 with TRAIL is involved in the activation of the p53-autophagy-DR5 signaling pathway in HeLa cells.

### 3.7. JNK Activation Is Involved in TRAIL/SNX-2112-Induced Apoptosis

It has been previously reported that by upregulating the level of ROS, drugs can activate the MAPK signaling pathway to overcome TRAIL resistance, sensitizing TRAIL-induced cancer cells to apoptosis. Here, we investigated whether SNX-2112 could induce MAPK in TRAIL/SNX-2112-induced apoptosis.

**Figure 7:**
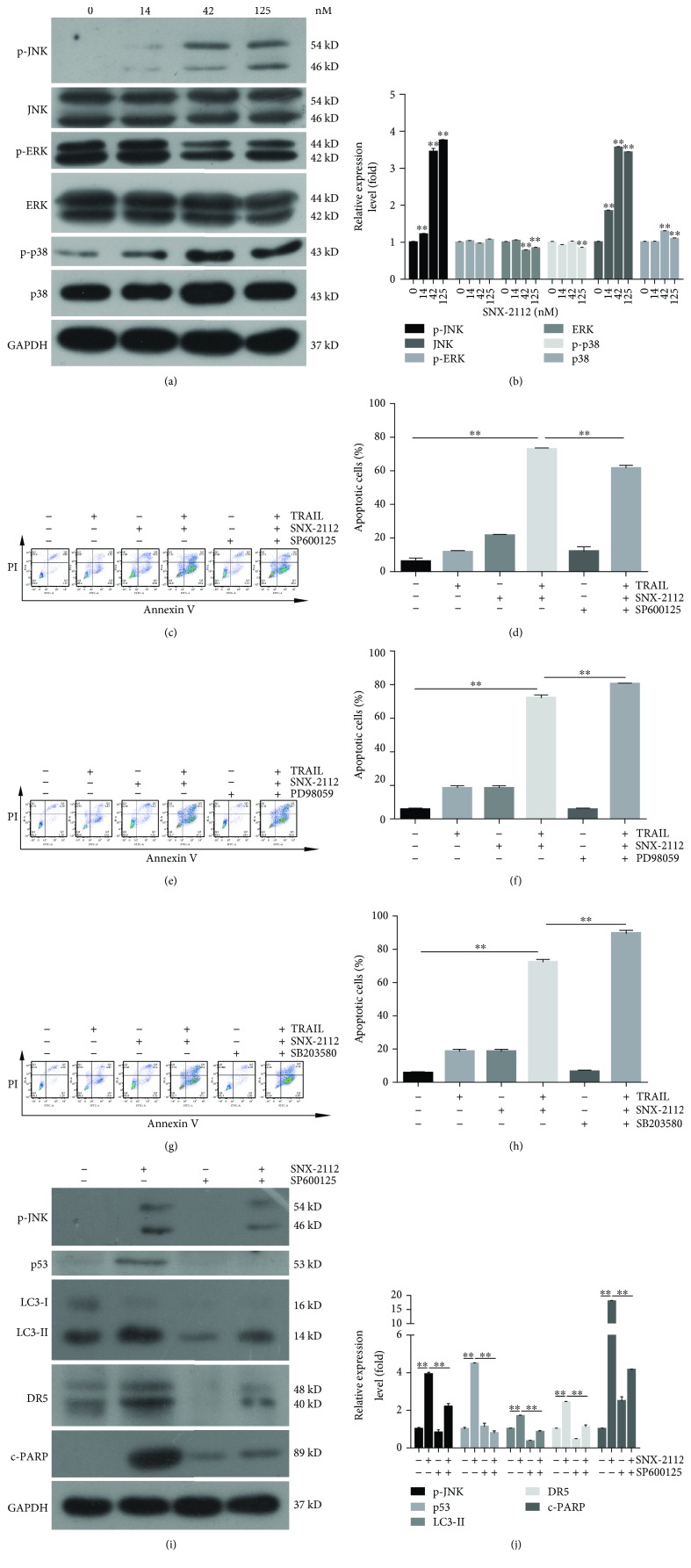
SNX-2112-induced JNK activation is involved in apoptosis induced by TRAIL/SNX-2112. (a, b) HeLa cells were treated with SNX-2112 at indicated doses for 6 h. Western blotting was performed to detect the levels of p-ERK, ERK, p-JNK, JNK, p-p38, and p38. Densitometry analyses of the bands for each protein were performed. HeLa cells were pretreated with (c, d) 10 *μ*M SP600125, (e, f) 25 *μ*M PD98059, and (g, h) 10 *μ*M SB203580 for 2 h and then treated with either TRAIL (200 ng/mL) or SNX-2112 (125 nM) alone or in combination for 48 h. TRAIL/SNX-2112-induced apoptosis was analyzed by flow cytometry. (i, j) HeLa cells were pretreated with or without 10 mM SP600125 for 2 h and then treated with or without SNX-2112 for 48 h. Western blotting were used to analyze whole-cell extracts and detect the levels of p53, LC3, and DR5. Densitometry analyses of the bands for each protein were performed. Data are represented as mean ± SD. Error bars represent SD from three separate experiments. ^∗^ *P* < 0.05 and ^∗∗^ *P* < 0.01 compared with the control group.

Western blotting was used to detect the expression of MAPKs, including extracellular signal-regulated kinases (ERKs), JNKs, and p38-MAPKs. The results showed that SNX-2112 markedly increased JNK and p38 phosphorylation, but slightly downregulated ERK phosphorylation, in a dose-dependent manner (Figures 7(a) and 7(b)). Downregulation of ERK by SNX-2112 may account, at least in part, for the synergistic effect.

HeLa cells were pretreated with the JNK inhibitor SP600125, p38 inhibitor SB203580, and ERK inhibitor PD98059. The results of the flow cytometry assay showed that SP600125 significantly reduced the apoptosis induced by a combination of SNX-2112 with TRAIL (Figures 7(c) and 7(d)). However, SB203580 and PD98059 did not abolish apoptosis induced by TRAIL and SNX-2112 (Figures 7(e)-7(h)). These results suggest that SNX-2112 can induce the activation of JNK to potentiate TRAIL-induced apoptosis in HeLa cells.

We further analyzed the relationship of JNK between the p53-autophagy-DR5 signaling pathways. As shown in Figures 7(i) and 7(j), JNK inhibitor SP600125 was applied to determine the role of JNK in the p53-autophagy-DR5 pathway. We detected the expression levels of p53, LC3, and DR5 in the presence of SP600125 by Western blotting in HeLa cells. The data showed that the upregulation of SNX-2112-induced DR5 was abolished, as with p53 and LC3-II. Thus, our results suggested that the apoptotic effect induced by the combination of SNX-2112 with TRAIL is induced by the activation of the JNK-p53-autophagy-DR5 signaling pathway in HeLa cells.

### 3.8. SNX-2112-Induced Accumulation of ROS Activates the JNK-p53-Autophagy Pathway to Mediate DR5 Expression

Many studies have confirmed that DR5 expression was induced by ROS generation [55, 56] and that autophagy-mediated cell death was mediated by ROS [57]. The effect of ROS on the potentiation of TRAIL-induced apoptosis by SNX-2112 was also investigated here.

**Figure 8:**
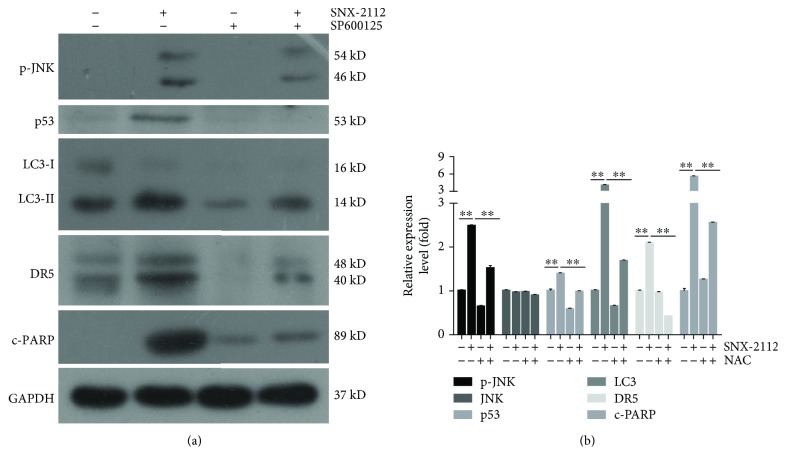
SNX-2112-induced ROS activates the JNK-p53-autophagy pathway to induce DR5 expression. HeLa cells were pretreated with or without 20 mM NAC for 2 h and then treated with or without SNX-2112 for 48 h. (a, b) Western blotting were used to analyze whole-cell extracts and detect the levels of p-JNK, JNK, p53, LC3, DR5, and c-PARP. Densitometry analyses of the bands for each protein were performed. Data are represented as mean ± SD. Error bars represent SD from three separate experiments. ^∗^ *P*<0.05 and^∗∗^ *P* < 0.01 compared with the control group.

To determine the role of ROS to JNK-p53-autophagy-mediated DR5 signaling, NAC, a reactive oxygen scavenger, was applied. In HeLa cells pretreated with NAC, we found that the upregulation of SNX-2112-induced DR5 was abolished, as with p-JNK, p53, LC3-II, and c-PARP (Figures 8(a) and 8(b)). SNX-2112-induced ROS activated the expression of p-JNK, p53, autophagy protein LC3-II, and death receptor DR5. Overall, it suggested that by increasing the level of ROS, SNX-2112 enhanced TRAIL-induced apoptosis via the JNK-p53-autophagy-mediated DR5 pathway in HeLa cells.

## 4. Discussion

TRAIL is a promising anticancer drug, but the resistance of cancer cells to this agent presents a huge obstacle for effective TRAIL-targeted therapy. As a result, agents that can either enhance the effect of TRAIL or overcome its resistance are needed. Many reports have shown that combination therapies produced a higher rate of apoptosis than did monotherapies in many cancers, including renal carcinoma [58] and human colon cancer [59]. In our study, we found that the Hsp90 inhibitor SNX-2112 enhanced TRAIL-induced apoptosis in human cervical cancer cells.

The Hsp90 complex mediates the maturation and stability of a variety of client proteins, many of which are crucial in oncogenesis, including Akt and mutant p53 [60]. Inhibition of Hsp90 function disrupts the complex and leads to degradation of client proteins in a proteasome-dependent manner. This results in simultaneous interruption of many signal transduction pathways pivotal to tumor progression and survival. It has been reported that the trial of silencing Hsp90 expression through transfection of Hsp90 siRNAs into HeLa cells could prevent the proliferation, the same as treating with the Hsp90 inhibitor 17-AAG [61]. Also, in lung cancer Calu-1 and H157 cells, inhibiting the Hsp90 expression by special siRNAs could reduce the expression of c-FLIPL [62, 63].

Although SNX-2112 has been previously found to be an anticancer agent against various cancer cells, its effect was generally limited. Our study first provided that the combination of SNX-2112 with TRAIL could enhance cancer cell death by downregulating antiapoptosis proteins, including Bcl-2, Bcl-XL, and FLIP, and elucidate the mechanism by which SNX-2112 sensitizes TRAIL to apoptosis in cervical cancer cells. At a mechanistic level, our results showed that SNX-2112 inhibited Akt/mTOR signaling to induce autophagy by the downregulation of Hsp90 client protein Akt (Figure 5) and upregulated the TRAIL-receptor DR5 (Figure 4) through the ROS-activated JNK-p53-autophagy singling pathway (Figures 3-8). We concluded that low concentrations of SNX-2112 allowed TRAIL to activate the death-receptor pathways.

It has been shown that when inhibiting the Hsp90 expression by special siRNAs, the expression of c-FLIPL was reduced in the cells, suggesting that c-FLIPL is a client protein of Hsp90, consistent with the downregulation induced by SNX-2112 (Figure 2(h)). 17-AAG, one of the Hsp90 inhibitors, could downregulate the expression of FLIP and, due to the inhibition of Hsp90, could recruit the CHIP to degrade the FLIP [62, 63].

In clinical studies, the downregulation of death-receptor expression has been one of the crucial causes of cancer cell resistance to TRAIL, and competitive antibodies targeting death-receptors are increasingly gaining attention. Understanding the role of DR5 in synergistic effect and the mechanisms of DR5 upregulation would be very informative. In colorectal cancer cells, tunicamycin was found to effectively enhance TRAIL-induced apoptosis through JNK-CHOP-mediated DR5 upregulation [59], and gefitinib enhanced TRAIL-induced apoptosis by autophagy- and JNK-mediated death-receptor upregulation [64]. Isoobtusilactone A sensitizes human hepatoma Hep G2 cells to TRAIL-induced apoptosis via ROS and CHOP-mediated DR5 upregulation [56]. Apigenin potentiates TRAIL therapy of non-small cell lung cancer via upregulation of DR4/DR5 expression in a p53-dependent manner [48]. These reports indicated that the drug-induced upregulation of death-receptor DR5 played a key role in the sensitization of TRAIL to cancer cells and demonstrated the great potential of DR5 in the development of antitumor drugs. Our results showed that SNX-2112 upregulated DR5 expression in HeLa cells in a concentration-dependent and time-dependent manner (Figures 4(d)-4(g)). Silencing of DR5 by DR5 siRNA significantly decreased cell apoptosis induced by SNX-2112 and TRAIL (Figures 4(j)-4(h)). This result was consistent with previous reports and indicated that death-receptor DR5 played a key role in TRAIL/SNX-2112-induced apoptosis. Our study showed for the first time that the upregulation of DR5 induced by SNX-2112 was the result of the activation of the ROS-p53-autophagy pathway. These results provide a novel strategy for oncological treatment, which is the combination of TRAIL with SNX-2112.

Both apoptosis and autophagy are essential cellular pathways related to degradation. They are induced by similar stimuli and regulated by similar pathways. Their crosstalk affects anticancer drug sensitivity and cell death [65]. However, the underlying mechanism remains unclear. It has been previously reported that autophagy and apoptosis cooperated to affect the fate of cells [66, 67]. Our previous study indicated that SNX-2112 could promote tumor cell death by inducing apoptosis and autophagy in human melanoma A-375 cells [36]. It was this same SNX-2112 that was found to induce autophagy by affecting the Akt/mTOR signaling pathway in HeLa cells in this study (Figures 5(a)-5(j)). The autophagy inhibitor BFA was found to effectively inhibit SNX-2112-induced cleavage of PARP, indicating that autophagy synergistically promoted apoptosis in HeLa cells (Figures 5(k) and 5(l)).

It has also been previously reported that TRAIL resistance was regulated by autophagy in some cells. However, the molecular mechanism of autophagy-mediated TRAIL resistance was not yet known [68]. The Guidelines by Klionsky et al. 2016 reported that lysosomal degradation can be prevented by protease inhibitors bafilomycin A1 and chloroquine, which could block the fusion of autophagosomes and increase the level of LC3-II [69]. It was recently found that, in the selected TRAIL-resistant cells (HepG2-TR), the inhibition of autophagy partially restored TRAIL-induced apoptosis and cytotoxicity [70]. However, in a study with colorectal cancer cell lines, the inhibition of autophagy by 3MA was found to significantly decrease DR4 and DR5 upregulation and reduce apoptosis [64]. These results indicated that autophagy might affect TRAIL resistance by regulating the expression of DR5, and our own results are consistent with these findings. We found that autophagy was induced by low concentrations of SNX-2112, and the inhibition of autophagy by BFA or Atg7 siRNAs significantly decreased upregulation of DR5 and the synergistic effect of TRAIL and SNX-2112 (Figure 5). Our findings provide an insight into the mechanism of action between SNX-2112-induced autophagy and TRAIL-mediated apoptosis.

The role of autophagy in DR5 regulation has been previously explored in human breast cancer cells. The data showed that high basal levels of autophagosomes sequestered DR4 and DR5 and effected cellular localization of death receptors, thought to contribute to their deficiency on the cell surface [49]. Also, the researchers speculated that bafilomycin A1 hinders the fusion of autophagosomes after apoptotic proteins are enclosed in lysosomes, in which the proteins were degraded. Hence, we considered the possibility that many apoptotic proteins are already trapped in the autophagosomes without being degraded during SNX-2112/TRAIL-induced apoptosis, which could not be stimulated to induce apoptosis. Same as our results, when the HeLa cells incubated with bafilomycin A1, DR5, a cell membrane receptor, was downregulated as shown in Figure 5(k).

From this, we hypothesized that drug-induced autophagy may play a different role than basal autophagy and that its function may be to act as an effective vector for death receptors or to protect and stabilize the structure of the death receptor. Further studies would be needed to determine the precise role of autophagy on death receptors.

As we all know, TRAIL has two receptors DR4 and DR5, and both can induce cell death in cancer cells. It has been reported that the two genes were transcriptional targets of p53 [71]. However, some cancer cells are not equally sensitive to TRAIL through the two receptors [72]. In accordance with these findings, our data showed that SNX-2112 could induce the expression of both DR4 and DR5, but only DR5 affects the apoptosis induced by a combination of TRAIL with SNX-2112 (Figure 4).

TRAIL receptors are differentially regulated. TRAIL differentially binds to TRAIL-R as illustrated by the phosphorothioate-modified CpG nucleotides, which block the binding of TRAIL to DR5 but not to DR4 [73]. TRAIL mutants with selective binding to DR4 or DR5 also showed that binding of TRAIL to DR4 and DR5 involved different amino acids [74].

SNX-2112 has been previously found to significantly decrease the expression of p53 and inhibit the proliferation of esophageal cancer cells [39]. As a target gene of p53, the gene transcription of DR5 is directly regulated by p53. Triptolide increased the level of DR5 in OCI-AML3, whereas increased DR5 was found to decrease in p53-knockdown OCI-AML3 and p53-mutated U937 cells, confirming the role of p53 in regulating DR5 expression [53]. The same phenomena were observed in myeloma cells [72], human hepatocellular carcinoma cells [75], prostate cancer cells [76], and human renal carcinoma cells [54]. Our results confirmed that SNX-2112-induced p53 could upregulate DR5 expression in cervical cancer HeLa cells. Interestingly, our results were the first to find that SNX-2112-induced p53 also conduced to the autophagy, contributing to a synergistic effect between SNX-2112 and TRAIL (Figure 6). This is a novel idea to explain the mechanism of action between p53 and DR5. Above all, our results suggest that the combination of TRAIL and SNX-2112 is more effective in cervical cancer cells with wild-type p53.

The detection of the MAPK signaling pathway proved that SNX-2112 activated TRAIL-induced apoptosis by activating JNK signaling in HeLa cells (Figure 7). The generation of drug-induced ROS has previously been found to activate JNK signaling in cancer cells, subsequently perturb the function of mitochondria, and result in mitochondria-related apoptosis in HeLa and other cancer cells [77–81]. Consistently, our results confirmed that SNX-2112-induced ROS upregulated p-JNK, following activation of the p53-autophagy-DR5 pathway.

One of the hallmarks of cancer is the deregulation of cellular energetics, causing a production of higher levels of ROS concomitant with alterations in antioxidant pathways [22]. Suffering from oxidative stress in the long term has made cancer cells acquire the adaptivity allowing them to survive in hypoxia and to become drug-resistant [82], so that, for different treatment strategies aiming at enhancing ROS production in cancer cells, it indicated a grand prospect leading to the selective killing of these cells when compared to normal cells [22]. There is much evidence that drug-induced ROS acting as an upstream signal can induce the upregulation of DR5 [77, 78]. Our results showed that ROS generation contributed to SNX-2112-induced upregulation of JNK-p53-autophagy-mediated DR5 (Figure 8), consistent with the induction of ROS production by other Hsp90 inhibitors [83]. The accumulation of ROS in HeLa cells is a striking feature of SNX-2112-induced apoptosis. Moreover, excessive ROS accumulation might play crucial roles in inducing both cell apoptosis and autophagy [57, 84]. Besides destabilizing Hsp90 client oncoproteins, SNX-2112 exerts cytotoxicity by generating ROS. There may be two explanations for this mechanism. Firstly, it has been reported that some Hsp90 inhibitors could induce mitochondrial ROS production by elevating endoplasmic reticulum (ER) stress originating from the accumulation of unfolded protein in the ER [21]. As a consequence of increased ER stress, mitochondrial homeostasis was disrupted and, thereby, the production of mitochondrial ROS was increased [21, 23]. Meanwhile, we have previously proven that SNX-2112 could elevate the role of ER stress to induce apoptosis [41]. Hsp90, a chaperone protein, regulates a variety of cellular processes by assisting in the folding of client proteins. Secondly, the *α*,*β*-unsaturated ketone moiety of SNX-2112 may interact with some oxidoreductases, such as thioredoxin reductase, and consequently cause the accumulation of ROS in cells [85–87].

**Figure 9:**
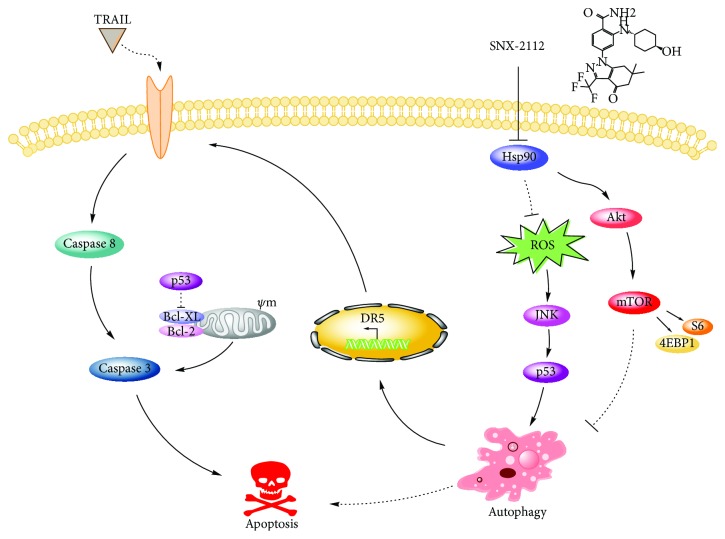
Illustration of the synergistic effect of SNX-2112 and TRAIL in HeLa cells.

In summary, our study is the first to demonstrate that SNX-2112 enhances TRAIL-induced apoptosis in HeLa cells through the ROS-mediated activation of JNK-p53-autophagy-DR5 (Figure 9). Our work contributes to further understanding the antitumor mechanism of SNX-2112. Most importantly, our results detailed the mechanisms of SNX-2112 in combination with TRAIL in cervical cancer cells, providing a reliable theoretical basis for the combination of TRAIL with Hsp90 inhibitor as a novel strategy for the treatment of human cervical cancer.

## Data Availability

The data used to support the findings of this study are available from the corresponding author upon request.

## Abbreviations

TRAIL: Tumor necrosis factor-related apoptosis-inducing ligand
Hsp90: Heat shock protein 90
DISC: The death-inducing signal complex
VEGFR2: Vascular endothelial growth factor receptor 2
ROS: Reactive oxygen species
Z-VAD-fmk: The cell-permeable pan caspase inhibitor
NAC: N-Acetyl-L-cysteine
BFA: Bafilomycin A1.

## Supplementary Materials

Supplementary Materials Supplemental Figure 1: the effect of SNX-2112 on the expression level of p62. HeLa cells were treated with SNX-2112 for the indicated (a-b) time (12, 24, and 48 h) or (c-d) concentration (0, 14, 42, and 125 nM). Western blotting was performed to detect the levels of p62. Densitometry analyses of the bands for each protein were performed. Data are represented as mean ± SD. Error bars represent SD from three separate experiments.
