# Supplementary material for: Hsp90 Inhibitor SNX-2112 Enhances TRAIL-Induced Apoptosis of Human Cervical Cancer Cells via the ROS-Mediated JNK-p53-Autophagy-DR5 Pathway
Source: Oxid Med Cell Longev. 2019 Mar 25;2019:9675450. doi: 10.1155/2019/9675450 (PMC6452544; doi:10.1155/2019/9675450)
Supplement: Supplementary Materials — Supplemental Figure 1: the effect of SNX-2112 on the expression level of p62. HeLa cells were treated with SNX-2112 for the indicated (a-b) time (12, 24, and 48 h) or (c-d) concentration (0, 14, 42, and 125 nM). Western blotting was performed to detect the levels of p62. Densitometry analyses of the bands for each protein were performed. Data are represented as mean ± SD. Error bars represent SD from three separate experiments. [file 9675450.f1.docx]

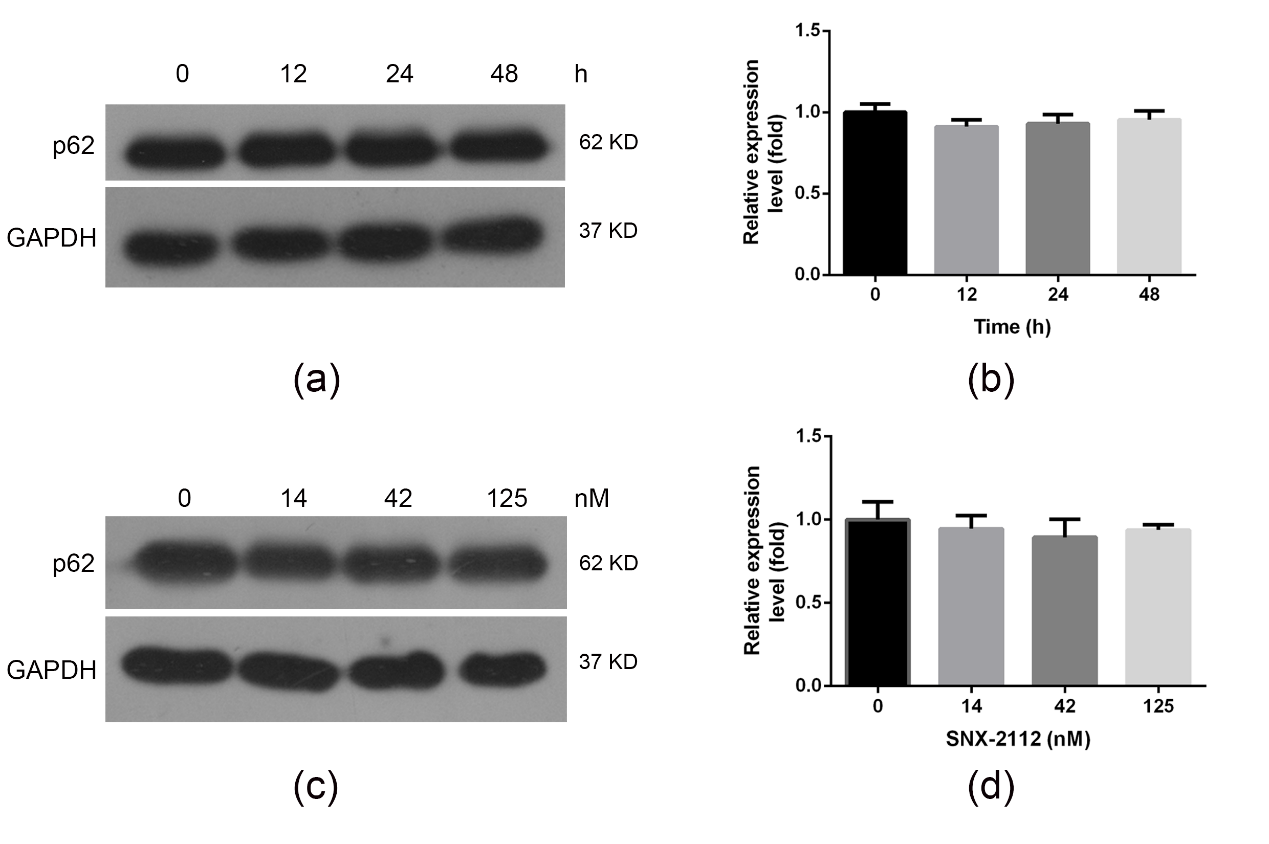


**Supplemental Figure 1:** **The effect of SNX-2112 on the expression level of p62.** HeLa cells were treated with SNX-2112 for the indicated (a-b) time (12, 24, 48 h) or (c-d) concentration (0, 14, 42, 125 nM). Western blotting was performed to detect the levels of p62. Densitometry analyses of the bands for each protein were performed. Data are represented as the mean ± SD. Error bars represent SD from three separate experiments.
